# Impact of Needle Selection on Survival of Muscle-Derived Cells When Used for Laryngeal Injections

**Published:** 2022-12-09

**Authors:** Oluwaseyi Awonusi, Zachary J. Harbin, Sarah Brookes, Lujuan Zhang, Samuel Kaefer, Rachel A. Morrison, Sharlé Newman, Sherry Voytik-Harbin, Stacey Halum

**Affiliations:** 1 Department of Otolaryngology-Head and Neck Surgery, Indiana University School of Medicine (IUSM), Indianapolis, IN, USA; 2 School of Mechanical Engineering, Purdue University, West Lafayette, IN, USA; 3 Weldon School of Biomedical Engineering, Purdue University, West Lafayette, IN, USA; 4 Department of Basic Medical Sciences, Purdue University, West Lafayette, IN, USA

**Keywords:** Autologous muscle-derived cells, Muscle progenitor cells, Cell viability, Injection needle, Stem cells, Biomaterials

## Abstract

**Objective:**

To describe how differing injector needles and delivery vehicles impact Autologous Muscle-Derived Cell (AMDC) viability when used for laryngeal injection.

**Methods:**

In this study, adult porcine muscle tissue was harvested and used to create AMDC populations. While controlling cell concentration (1 × 10^7^ cells/ml), AMDCs including Muscle Progenitor Cells (MPCs) or Motor Endplate Expressing Cells (MEEs) were suspended in either phosphate-buffered saline or polymerizable (in-situ scaffold forming) type I oligomeric collagen solution. Cell suspensions were then injected through 23- and 27-gauge needles of different lengths at the same rate (2 ml/min) using a syringe pump. Cell viability was measured immediately after injection and 24- and 48-hours post-injection, and then compared to baseline cell viability prior to injection.

**Results:**

The viability of cells post-injection was not impacted by needle length or needle gauge but was significantly impacted by the delivery vehicle. Overall, injection of cells using collagen as a delivery vehicle maintained the highest cell viability.

**Conclusion:**

Needle gauge, needle length, and delivery vehicle are important factors that can affect the viability of injected cell populations. These factors should be considered and adapted to improve injectable MDC therapy outcomes when used for laryngeal applications.

## INTRODUCTION

Cellular therapies in laryngology are advancing and have shown promising clinical benefits. Clinical application of these cellular therapies include, but are not limited to, the use of autologous fibroblasts to treat vocal fold scarring [1], and Autologous Muscle-Derived Cells (AMDCs) in the treatment of dysphagia [2]. Recent clinical trials by Plowman, et al. have suggested that AMDCs have the potential benefit of improving tongue function in patients with oropharynx cancer [2]. AMDCs are of particular interest in diseases resulting in loss of muscle function due to their contribution to volumetric muscle growth and regeneration [3]. Studies by Halum, et al. in preclinical animal models have induced AMDCs to express motor endplates; these AMDC-derived cells are called Motor Endplate-Expressing Cells (MEEs) [4,5]. MEEs have been shown to express neurotrophic factors that, when delivered into denervated muscle, can promote target muscle reinnervation [4-6]. MEEs have thereby been shown to be a promising treatment for laryngeal denervation injury [6].

The therapeutic potential of Muscle-Derived Cellular Therapies (AMDCs and MEEs) depends on the successful implantation of viable cells through a needle while preserving cell function [7]. Since AMDC and MEE therapies are commonly delivered through needles, cells are exposed to various elements during the delivery process that can damage the cell leading to death/apoptosis and hampered therapeutic benefit [8]. Certain parameters such as needle size, cell seeding density, delivery vehicle biomaterial, ejection flow rate [7], and cryopreservation methods [9] have all been shown to impact cell viability and efficacy of cellular therapies. Most studies that have investigated the impact of delivery parameters on cell viability have focused on Mesenchymal Stromal Cells (MSCs). In fact, there are no previous investigations demonstrating the impact of needle selection on AMDC and MEE cell viability. During AMDC and MEE injection, shear stress from mechanical forces during the delivery process is reported to be one of the key elements accounting for cell damage [7]. Shear stress is influenced by the needle bore size, the cell size and shape, and the delivery biomaterial characteristics. Some studies reported no effect of narrow-bore needles on the viability of MSCs [8-10], while others report cell viability to be negatively impacted with needles of small diameter [7,11,12]. Similarly, there are also varied reports on how the characteristics of the biomaterial being delivered impacts cell viability [7-9]. Some studies have reported that more viscous biomaterials such as hydrogels protect cells from biomechanical stress during delivery [9] while others suggest that high viscosity increases the shear forces [7]. These studies on MSCs cannot be reliably applied to AMDCs and MEEs due to differences in cell sizes and other characteristics.

The purpose of this study was to investigate the impact of commonly used laryngology needles of different diameters and lengths on the survival of porcine AMDCs and MEEs. Viability, apoptosis and phenotypic expression were measured after ejection when cells were suspended within either phosphate-buffered saline (PBS) or polymerizable type I oligomeric collagen (7.51 mg/ml) while controlling for a constant flow rate (2 ml/min) and cell density (1 × 10^7^ cell/ml). As AMDC and MEE therapies translate to clinical trials, findings from this investigation will help guide optimization of the delivery process in clinical care models.

## MATERIALS AND METHODS

### Autologous muscle- derived cell culture

Autologous Muscle- Derived Cells (AMDCs) were isolated from skeletal muscles of Yucatan minipigs (S&S Farms, Malta, IL). Cells were cultured for two to three passages in tissue culture treated flasks (Corning Life Sciences, Corning, NY) in Dulbecco’s Modified Eagle Medium (DMEM) supplemented with 20% fetal bovine serum (FBS, Hyclone, Logan, Utah), 1% penicillin, streptomycin, and amphotericin B (PSF-1, Hyclone, Logan, Utah). Cells were incubated with 5% CO2 at 37°C with media changes every 2 days. At 80% confluency cells were detached from the flask using trypsin, centrifuged and used for the study.

### Motor endplate-expressing cell differentiation

Cultured AMDCs were allowed to reach confluence. Cells were then cultured with differentiation media containing DMEM supplemented with 2% horse serum and 1% PSF-1 for 5 days with media changes every 48 hours. Thereafter, induction media containing agrin (10 nM, R&D systems), neuregulin (2 nM, R&D Systems), and acetylcholine (10 nM, R&D Systems, Minneapolis, MN) was added to induce motor endplate formation. After 5 days in induction culture media, the cells were used for the study. MEE differentiation was confirmed by immunostaining with Alexa Fluor 594 conjugated bungarotoxin (Molecular Probes, Eugene, Oregon) [6].

### Injection of cells

AMDCs and MEEs reconstituted to a concentration of 1 × 10^7^ cells/ml were suspended in either PBS or type I oligomeric collagen (OM10027, GeniPhys, Zionsville, IN) in preparation for injection. Cells were loaded into a 1ml BD syringe (Becton, Dickinson and Co, Franklin Lakes, NJ) with attached selected needle: 22G (1.5 and 3.5 in; Exelint International Co), 27G (1.5 and 3.5 in; Exelint International Co, B. Braun Medical Inc.), 23G William Cook (17.7 in, Cook Medical), and 27G Oro-tracheal needle (9.5 in, Integra Lifesciences, Princeton, NJ) (Table 1). The syringe-needle combination was injected with a NE-500 programmable syringe pump (New Era Syringe Pump Inc.) at a flowrate of 2 ml/min into a 0.6 ml Eppendorf tube. Of note, the 27G Oro-tracheal needle injection was performed manually as it could not be manipulated to fit the syringe pump. Ejected volume for all the cell samples was 0.5 ml (n=3 separate trials). At the start of each trial, control samples were collected using a 200 μL pipette. For Immediate Cell Viability testing, cell viability was measured immediately after pipette (control) or needle ejection (study group) in all samples. For the Temporal Cell Viability testing, cells from each PBS-suspended sample were incubated with serum-deprived DMEM for 24 or 48 hours post-ejection to mimic the transient serum deprivation conditions that cells may experience after *in vivo* injection. After live/dead staining (see Cell Viability Assay below), the cells were fixed with 4% formaldehyde or 20 minutes and then imaged with a Zeiss LSM 880 confocal microscope (Oberkochen, Germany). For all the collagen suspended cell ejection Viability Assay trials, the collagen-cell suspension was ejected through the pipette (control) or needle (test group), allowed to polymerize, and then incubated with serum-deprived DMEM for 24 or 48 hours. After live/dead staining (see Cell Viability Assay below), the 3D collagen-cell culture was fixed with 4% formaldehyde for 20 minutes and then imaged with a Zeiss LSM 880 confocal microscope (Oberkochen, Germany).

### Biophysical and biomechanical injection characteristics

**Figure 1:**
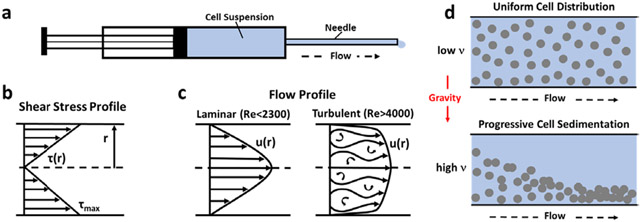
Schematics detailing biomechanical concepts relevant to injection of cell suspensions through syringe-needle systems. (a) Diagram of the syringe-needle system showing flow path through needle with inner radius r. (b) Shear stress profile (τ(r)) as occurs within needle during injection. Maximum Shear Stress $\left(\tau_{\max}\right)$ occurs at the needle wall, decreasing linearly as the center axis of the cylindrical needle is approached. (c) Reynold’s number (Re) is used to predict laminar or turbulent flow profiles, which exhibit unique velocity profiles (u(r)) with respect to needle dimensions. (d) Sedimentation velocity v, which is largely dependent on and inversely related to dynamic viscosity of the delivery vehicle, determines cell distribution throughout the injection process. Cell sedimentation during injection contributes to inconsistent dosing, unpredictable cell distribution, and clogging of needle.

Dynamic Viscosity, Shear Stress, Flow Profile, and Sedimentation. Dynamic viscosity of a delivery vehicle or cell suspension describes the force needed to overcome the internal molecular friction so that the solution or suspension will flow (Figure 1a). The dynamic viscosity of the PBS vehicle alone was determined to be 0.92 × 10^−3^ kg/(m.s), which is consistent with previously published values [7]. Dynamic viscosity of the polymerizable collagen solution was 49.7 × 10^−3^ kg/(m.s), as measured at a shear rate consistent with that of the injection. Based on these values, the dynamic viscosity of cell suspensions within each vehicle was calculated according to the Krieger-Dougherty equation

$$\eta_{s}=\eta_{v}(1-\frac{\phi }{\phi_{m}}{)}^{-\eta_{i}\phi_{m}}$$

where $\eta_{s}$ (kg/(m.s)) is the dynamic viscosity of the cell suspension within a specific vehicle, $\eta_{v}$ (kg/(m.s)) is the dynamic viscosity of the vehicle alone, φ is the volume fraction of cells in the suspension, $\varphi_{m}$ is the maximum volume fraction of the cells in the suspension, and $\eta_{i}$ is the intrinsic viscosity of the medium. Assuming that cells act as spherical particles in suspension, a value of 2.5 was applied for $\eta_{i}$. Additionally, $\varphi_{m}$ was assigned a value of 1 to represent a cell suspension with no vehicle. Further φ was calculated by dividing the cell concentration 1 × 10^4^ cells/μL by the maximum concentration of cells 2 × 10^5^ cells/μL, which was calculated based on published cell volume measurements of 5000 μm^3^ [7,13].

Shear stress describes the drag force per unit area imposed by the vehicle or cell suspension in motion on the needle wall in parallel to the direction of flow (Figure 1b). The shear stress of the cell suspension, when delivered in PBS or the polymerizable collagen solution, was calculated for each needle size at the applied flow rate of 2 ml/min according to the Law of Poiseuille:

$$\tau_{\max}=\frac{4Q\eta_{s}}{\pi R^{3}}$$

Where $\tau_{\max}$ (N/m^2^) is shear stress, Q (m^3^/s) is the volumetric flow rate, $\eta_{s}$ (kg/(m.s)) is the dynamic viscosity of the cell suspension, and R (m) is the inner radius of the needle. Reynold’s number (Re), which is a dimensionless parameter used to predict flow patterns, was calculated as follows:

$$R_{e}=\frac{\rho Q}{15\pi D\eta_{s}}$$

Where ρ (kg/m^3^) is the density of the vehicle, Q (m^3^/s) is the volumetric flow rate, D (m) is the needle diameter and $\eta_{s}$ (kg/(m.s)) is the dynamic viscosity of the cell suspension. The density of the PBS and collagen solutions were measured to be 1000 kg/m^3^ and 999 kg/m^3^, respectively. The flow profile is said to be laminar for Re<2300 and turbulent for Re>4000 (Figure 1c). Transitional flow occurs for Re values between 2300 and 4000. Cells within a suspension that is contained within a syringe or needle may sediment or settle out of the fluid due to gravitational forces acting on them (Figure 1d). As such, cell sedimentation causes in homogeneities within the injected cell suspension. For calculation of the sedimentation velocity (n; (m/s)) of cells in PBS and collagen, Stoke’s law was used:

$$v=\frac{2(\rho_{c}-\rho )d^{2}g}{9\;\eta_{v}}$$

where d (m) is the cell diameter, $\eta_{v}$ (kg/ (m.s)) is dynamic viscosity of the vehicle, $\rho_{c}$ (kg/m^3^) cell density, r (kg/m^3^) is the vehicle density, and g is the gravitational constant. For these calculations, values of 21.2 × 10^−6^ m and 1020 kg/m^3^ were used for d and rc, respectively.

### Cell viability assay

Cell viability was assessed using Acridine Orange/Propidium Iodide (AO/PI) (Togos biosystems, Gyeonggi-do, South Korea) with an automated image-based cell counter (LUNA-FX7TM Togos biosystems, Gyeonggi-do, South Korea). Acridine orange and propidium iodide stains bind to double- stranded DNA and determines cell viability based on the integrity of the cell membrane. Propidium iodide emits red fluorescence (excitation/emission: 533/617 nm) in dead cells without intact plasma membranes. In comparison, acridine orange dye easily permeates intact membranes of viable cells and emits green fluorescence (excitation/emission: 500/526 nm) when bound to double- stranded DNA. The fixed cells were imaged on a Zeiss LSM 880 confocal microscope (Oberkochen, Germany) at 20X. Three images at random for each well plate were captured and analyzed with Image J. This cell viability was measured immediately after injection and at 24- and 48-hours post-injection.

### Apoptosis assay

Apoptosis was determined using Fluorescein Isothiocyanate-Conjugated (FITC) Annexin V and PI kit (Thermos Fisher Scientific, Eugene, OR). Normal live cells have Phosphatidylserine (PS) positioned on the inner leaflet of the plasma membrane. One of the hallmarks of early apoptosis is the translocation of PS to the outer leaflet of the plasma membrane. The exposed PS becomes available to bind Annexin V, a protein that binds PS with high affinity [14]. PI stains dead cells, as previously mentioned. Annexin V positive cells emit green fluorescence (excitation/emission: 488/518 nm). To induce apoptosis, cells were treated with 1 μM staurosporine, a protein kinase inhibitor (Abcam, Waltham, MA), and incubated for 4 hours which served as a positive control sample. According to the manufacturer’s recommended protocol, Annexin V/PI was added to cells and incubated in the dark for 15 minutes, then analyzed immediately with a flow cytometer (Bigfoot2 Cell Sorter, Invitrogen, Waltham, MA). 10,000 events were collected for each cell sample. The flow cytometry data was subsequently analyzed using FCS express 7 software to compare apoptotic fractions in control and needle ejected cells.

### Statistical analysis

All statistical analysis and graphing were performed using Graph Pad Prism 9 GraphPad Software, San Diego, CA. Data were Analyzed Using One- And Two-Factor Analysis Of Variance (ANOVA) followed by a Kruskal-Wallis test to determine the differences in viability between the needles tested. A critical global p-value ≤ 0.05 was considered statistically significant.

## RESULTS

The mean cell size of AMDCs used in this study was (18.53 μm ± 1.10) and MEEs (20.79 m ± 1.70). The maximum shear stress values for the various needles and delivery vehicles used were calculated (Table 2).

### Biophysical and biomechanical injection characteristics

To better define how needle size and delivery vehicle affect the biophysical and biomechanical characteristics of cell delivery, Maximum Shear Stress (t_max_) and Reynold’s Number (Re) were determined for the various injection conditions as summarized in Table 2. In general, shear stress values increased as needle size decreased, with cell suspensions in PBS ranging from 5.1 N/m^2^ for 22G needles to 38.5 N/m^2^ for smaller 27G needles. The dynamic viscosity of collagen was roughly 50 times greater than PBS, contributing to higher shear stress values ranging between 272.3 N/m^2^ and 2071.4 N/m^2^. For both delivery vehicles, the majority of the shear stress values were above those reported for blood flowing through arteries (1-7 N/m^2^) and veins (0.1-0.6 N/m^2^) [14, 15]. Interestingly, shear stress values reported for other cell suspension injection studies range from 0.424 N/m^2^ to 384 N/m^2^ [13, 16]. It is noteworthy that shear stress is also directly related to flow rate, so a reduction in flow rate from 2 ml/min to 0.2 ml/min would yield a 10-fold decrease in shear stress values.

The flow profile of the cell suspension during injection is dependent upon the vehicle density, cell suspension viscosity, needle diameter, and flow rate, with terms in the numerator and denominator of the Re equation contributing to inertial and viscous forces, respectively. More specifically, decreasing needle size yielded an increase in Re from 1.63 to 3.21 for cells in PBS and 0.030 to 0.060 for cells in collagen. The magnitude difference in Re values for cells suspended in PBS and collagen is largely owing to differences in vehicle dynamic viscosity. With the corresponding vehicle densities being roughly similar, the decreased viscosity of PBS results in a greater Re compared to that of the collagen. For all conditions, Re was less than 2300, indicating laminar or streamline flow (Figure 1c). Under these conditions, fluid layers and associated cells flow in parallel over one another with essentially no mixing between laminas. Given that sedimentation velocity is largely dependent upon and inversely related to the dynamic viscosity of the vehicle, values calculated for collagen (4.14 × 10^−7^ m/s) were roughly 50 times lower than those for PBS (2.13 × 10^−5^ m/s), indicating that the collagen assists in maintaining a homogenous cell suspension and improving consistency in cell delivery dose.

### Cell viability in AMDCs after injection

To examine the influence of needle type on AMDCs cell survival we injected these cells with a constant flow rate (2 ml/min) and concentration (1 × 10^7^ cells/ml).

### Immediate cell viability

There was no immediate change in cell viability after needle ejection across the different needles. Cell viability remained at >85% when measured immediately after each ejection.

### Temporal cell viability

**Figure 2:**
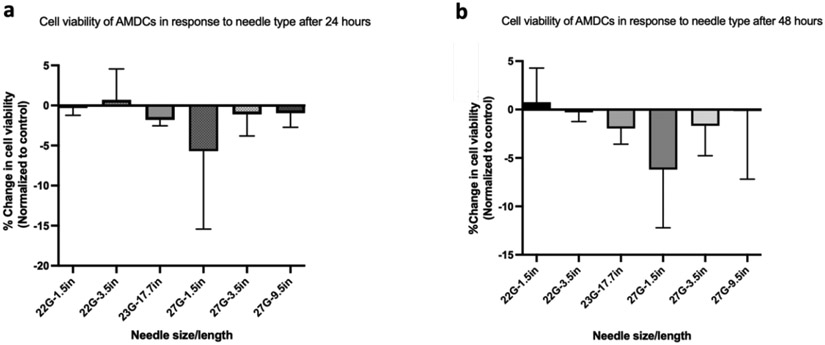
Percentage change in porcine autologous muscle derived cells (AMDCs) cell viability in saline following injection. Live/dead staining with propidium iodide/acridine orange was used to determine cell viability. (a) Change in cell viability of AMDCs at 24hours and (b) 48 hours following injection through various needles of different diameter and length. There is no difference in the change in cell viability across all the needles tested. Data were normalized to the control within each group (two-way Analysis of Variance (ANOVA) with Kruskal-Wallis’ test), n=3.

At 24- and 48-hours post-needle ejection, both control and all needles’ groups had a significant loss of surviving cells compared to the starting time point (the immediate cell viability). The reduction in survival in both treated and untreated groups was secondary to the serum-deprived culture medium which was used to closely mimic the cellular stress experienced after *in vivo* injection. After normalizing the groups at the aforementioned time points, data analysis showed no difference in cell viability between the needles. Figure 2 shows the cell viability over time based on the needles. AMDCs suspended in type I collagen maintained viability close to 100% at all time points and were unaffected by the needle type (Table 3).

### Cell viability in MEEs after injection

**Figure 3:**
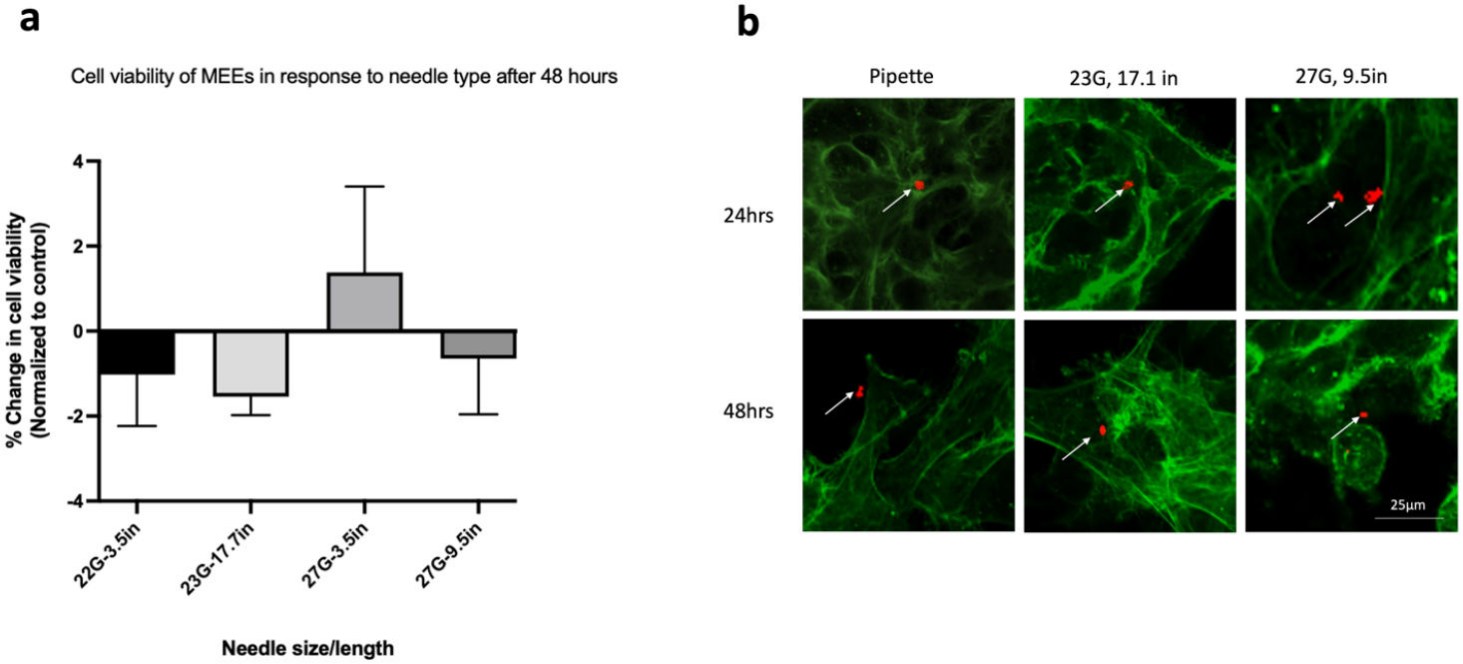
Percentage change in cell viability and phenotypic expression of Motor Endplate Expressing Cells (MEEs) following injection. (a) No difference in the change in cell viability across all needle types. Data was normalized to the control within the same group and compared between needles (two-way Analysis of Variance (ANOVA) with Kruskal-Wallis’ test), n=3 (b) Arrows depicting the presence of motor endplates after conjugated antibody α-bungarotoxin staining (red fluorescence) at 24- and 48-hours post-injection through 23 and 27G needles. Note: Cell cytoskeleton stained with F-actin (green fluorescence); scale bar= 25μm.

To assess the effect of needle type on the survival of MEEs, MEEs were injected with the same delivery parameters as previously detailed. Consistent with the ADMC findings, Immediate Cell Viability was >85% after needle ejection. Also consistent with ADMC Temporal Cell Viability findings, at 24 and 48 hours post-needle ejection, cell viability decreased in both control and treated samples. When data were normalized to the control, no difference in cell viability was detected across the needle types or across time as shown in Figure 2. To investigate the effect of injection delivery on motor endplate expression, control and treated cells were stained with Alexa Fluor 594 conjugated α-bungarotoxin (which is expressed at neuromuscular junctions) at 24- and 48-hours to detect the presence of acetylcholine receptors. Only the 23 G (17.7 in) and 27 G (9.5 in) needles were used for this study. As shown in Figure 3, red staining confirms the presence of motor endplates in both control and injected cells at 24 and 48 hours. There was no observable difference in the expression of motor endplate based on needle type.

### Apoptosis in AMDCS after injection

**Figure 4:**
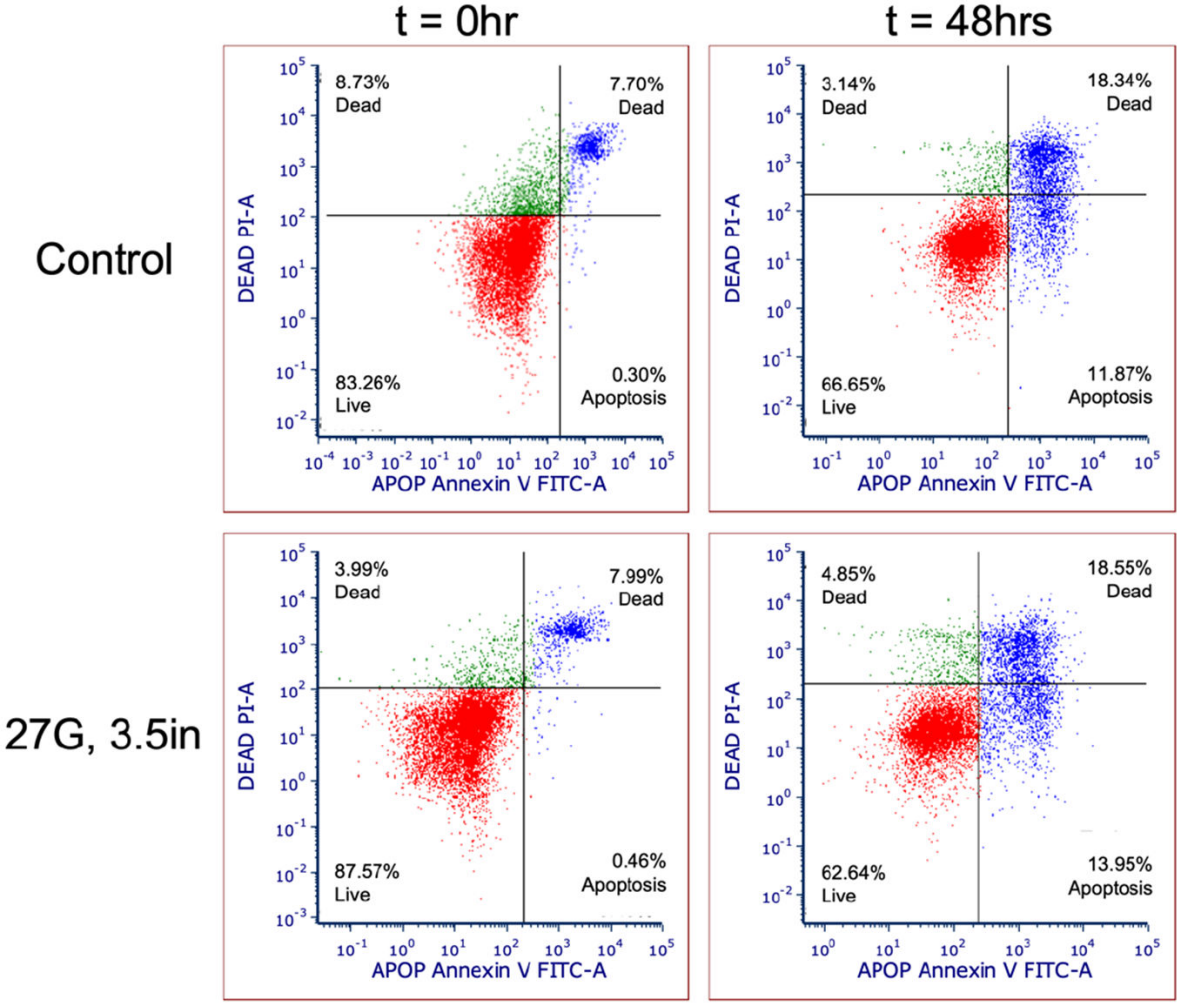
Representative flow cytometry dot plot diagrams showing apoptosis after staining with Annexin V-FITC and PI. AMDCs were stained immediately after injection (0 hr) and at 48 hrs; control cells were not injected. **Note :** Red dots in the left lower quadrant (Annexin V−/PI−) represent healthy live cells; blue dots in the right lower quadrant (Annexin V+/PI−): early apoptotic cells, green dots in the left upper quadrant (Annexin V−/PI+): dead cells, and blue dots in the right upper quadrant (Annexin V+/PI+): late apoptotic/dead cells).

The proportion of apoptotic cells in cell suspensions injected was comparable between 22-, 23- and 27-gauge needles of varying lengths based on flow cytometry data. There was a demonstrated increase in apoptotic cells after 48 hours in both needle ejected and control cells, consistent with the increased number of dead cells viewed in cell viability staining. Table 4 shows the average apoptotic percentage of cells immediately and 48 hours after injection. Data analysis detected no statistically significant difference in the proportion of apoptotic cells between the needle sizes and when compared to the control. The flow cytometry dot plot shown in Figure 4, illustrates that cell populations are categorized, based on staining, including healthy viable cells (double negative for Annexin V and Propidium Iodide), early apoptotic cells (only Annexin V positive), and late apoptotic and necrotic or dead cells (double-positive) [8,17]. During early apoptosis, the cell membrane is intact, preventing impermeable PI from staining cells; therefore, Annexin V positive only cells indicate early apoptosis. However, during the late stages of apoptosis and necrosis, the cell membrane is disrupted, allowing PI to penetrate; hence double-positive staining of Annexin V/PI indicates late apoptosis. Notably, Annexin V/PI staining cannot distinguish between late apoptotic and necrotic cell populations [17].

## DISCUSSION

As muscle-derived cellular therapies translate to clinical application, the need to understand the impact of the delivery process on the health of the transplanted cells has become increasingly important. This study evaluated the cellular health of AMDCs and MEEs following injection with different needle sizes and suspension vehicles. We demonstrated that needle selection has no considerable influence on the viability of AMDCs and MEEs. Furthermore, we found the delivery vehicle was an essential factor to consider in maintaining cell viability.

We measured cell viability and apoptosis after injection through different needle sizes and lengths: 22G, 23G, 27G, with lengths ranging from 1.5 inch to 17.7 inch. Long and narrow bore needles are preferred for easy and precise access to laryngeal muscles clinically, and to minimize patient discomfort (when injections are performed under local anesthesia) [9]. The needles were selected to reflect current clinical practice for cell therapies in laryngology; however, findings are relevant to other fields using ADMCs in clinical trials, such as urology [18]. A syringe pump was used to standardize the flow rate. Cell viability measured immediately after injection is typically an inadequate predictor of cell death as membrane damage may be delayed [7]. Therefore, viability was measured at prolonged time points (24 and 48 hours). We maintained a flow rate of 2 ml/min for all injections, which is higher than the rates used in some studies (1–300 1/min). [7,11] Some studies have reported that slower flow rates increase the number of cells left behind in the syringe and needle [16]. We chose this higher flow rate to better mimic the rate with which laryngologists inject material into the larynx. This study demonstrated that when controlling for cell density and flow rate, cell membrane integrity disruption and apoptosis of AMDCs and MEEs did not significantly vary across the different needle types. Neither the needle diameter nor the length had an impact on cell survival. To further examine the effect of needle size on phenotypic expression of differentiated Motor Endplate Expressing Cells (MEEs), the cells were stained for motor endplate markers following injection through 23 and 27G needles. It was observed that the injection needle had no impact on the phenotypic expression of motor endplates as shown by the presence of these motor endplate markers. As motor endplate expression is associated with the release of an array of neurotrophic factors, these findings suggest preserved function of the differentiated cells post-injection.

Stem cell therapies are often distributed in delivery biomaterials such as hyaluronic acid and collagen-based injectable material [19]. These biomaterials are selected for their potential to provide a biocompatible environment for cell adherence, growth, and cell encapsulation for barrier protection. However, the viscosity of these hydrogels is significantly higher than PBS or saline. Studies have shown that the viscosity of cell suspension is directly proportional to the shear stress; therefore, highly viscous fluids can potentially affect the cellular response of these stem cells during delivery [7]. In this study, we evaluated the cellular response regarding the cell viability of AMDCs in type 1 oligomeric collagen. It was observed that collagen sustained cell viability over PBS alone. Although cell-collagen suspension had the highest stress value, cell viability remained unaffected by needle size or length. Injection of collagen-cell suspensions with smaller bore needles (27G) produced high shear stress (2071.4 N/m^2^) when compared to in cell-PBS suspensions in larger bore needles (22G; 5.1 N/m^2^). Our data showed that cells suspended in collagen maintain high viability (96 to 100%) even after 48 hours of serum deprivation. Although the cell-collagen suspensions had significantly higher shear stress, it was insufficient to cause significant cell death. Thus, type 1 collagen in its oligomeric form may be a favorable delivery vehicle for AMDCs. The maintenance of cell viability in type 1 collagen is consistent with previous studies by Stephens, et al. [20] highlighting the influential properties of type 1 oligomeric collagen in sustaining the survival of 3D cultured islet cells. This phenomenon can be explained as type 1 oligomeric collagen has abundant binding sites for integrin, which mediate cell adhesion and signal transduction. As such, collagen provides a favorable environment for cell-matrix interaction promoting cell growth, function, and proliferation [19-21].

## CONCLUSION

Our study highlights potential parameters to consider during the injection of cellular therapies. The manipulation of Autologous Muscle-Derived Stem Cells (AMDCs) and Motor Endplate Expressing Cells (MEEs) with narrow bore size needles (as small as 27 gauge) did not affect cell viability, apoptosis or phenotypic characteristics. This study shows that cells injected with polymerizable (*in situ* forming) type 1 oligomeric collagen showed greater survival than cells injected with PBS when kept in nutrient-deprived conditions over time (48 hours). Thus, this data indicates that delivery vehicle selection maybe an important factor in the clinical success of cellular therapies.

## Figures and Tables

**Table 1:** Inner diameter (mm) and length (inches) of various needle types.

Needle type	Catalog number	Gauge	Inner diameter (mm)	Length (inches)
Hypodermic needle	210110	22	0.413	1.5
Spinal needle	26967	22	0.413	3.5
William’s cystoscopy needle	13828985	23	0.337	17.7
Hypodermic needle	201122	27	0.21	1.5
Spinal needle	S2735	27	0.21	3.5
Oro-tracheal injector (Integra ENT)	1650025	27	0.21	9.5

**Table 2:** Cell suspension dynamic viscosity and maximum shear stress values for various needle size and delivery vehicle.

Gauge	Inner diameter (mm)	Delivery vehicle	Suspension dynamic viscosity (kg/m.s)	Shear stress (N/m^2^)	Reynold’s number	Flow profile
22	0.413	PBS	0.00105	5.1	1.63	Laminar
		Collagen	0.0565	272.3	0.03	Laminar
23	0.337	PBS	0.00105	9.3	2	Laminar
		Collagen	0.0565	501.2	0.037	Laminar
27	0.21	PBS	0.00105	38.5	3.21	Laminar
		Collagen	0.0565	2071.4	0.06	Laminar

**Table 3:** Cell viability of AMDCs in PBS and collagen following injection with 22 and 27G needles immediately after injection, at 24 and 48 hours.

Needle gauge/length	Delivery vehicle	0 hr. viability (%)	24 hr. viability (%)	48 hr. viability (%)
22G, 3.5in	Collagen	97.27 ± 1.9	100	99.5 ± 0.5^a^
	PBS	86.9 ± 4.6^a^	77.9 ± 6.9^a^	70.8 ±10.8^a^
27G,3.5 in	Collagen	100	100	100
	PBS	89.4 ± 3.2^a^	80.3 ± 6.2^a^	70.0 ± 10.8^a^
27G, 9.5in	Collagen	100	100	100
	PBS	96.9 ± 1.5^a^	93.4 ± 19.6^a^	82.4 ± 8.1^a^

**Note:** Cell-collagen suspensions maintained high viability compared to cell-PBS suspensions, showing progressive cell loss at 24 and 48 hours. Mean ± SEM, n=3

a represents a significant difference from collagen treated group, p< 0.05.

**Table 4:** Porcine Autologous Muscle Derived Cells (AMDC) early apoptotic cell percentage (%) immediately after injection (0hr) and at 48 hrs post needle ejection through various needles.

Needle size	t=0 hr	t=48 hrs
22G, 3.5 in	0.5 ± 0.09	16.29 ± 4.02
23G, 17.7 in	0.6 ± 0.03	15.38 ± 1.07
27G, 3.5 in	0.49 ± 0.07	12.87 ± 3.86
27G, 9.5 in	0.62 ± 0.05	20.84 ± 8.75
Control	0.41 ± 0.12	12.63 ± 1.95

**Note:** No statistically significant difference in apoptosis of cells was detected across needles when compared to control samples. (Analysis of variance with Kruskal-Wallis test p< 0.05).
